# Acute Kidney Injury and Urinary Biomarkers in Human Immunodeficiency Virus–Associated Cryptococcal Meningitis

**DOI:** 10.1093/ofid/ofx127

**Published:** 2017-06-20

**Authors:** Charlotte Schutz, David R. Boulware, Katherine Huppler-Hullsiek, Maximilian von Hohenberg, Joshua Rhein, Kabanda Taseera, Friedrich Thienemann, Conrad Muzoora, David B. Meya, Graeme Meintjes

**Affiliations:** 1 Department of Medicine, Faculty of Health Sciences, and; 2 Clinical Infectious Diseases Research Initiative, Institute of Infectious Disease and Molecular Medicine, University of Cape Town, South Africa; 3 Division of Infectious Diseases & International Medicine, Department of Medicine, and; 4 Division of Biostatistics, School of Public Health, University of Minnesota, Minneapolis; 5 Infectious Disease Institute, Makerere University, Kampala; 6 Mbarara University of Science and Technology, Mbarara; 7 Makerere University College of Health Sciences, School of Medicine, Kampala, Uganda

**Keywords:** acute kidney injury, amphotericin B, biological marker, cystatin C, neutrophil gelatinase-associated lipocalin, protein, tissue inhibitor of metalloproteinase-2

## Abstract

**Background:**

*Cryptococcus* is the most common etiology of adult meningitis in Africa. Amphotericin B deoxycholate remains paramount to treatment, despite toxicities, including acute kidney injury (AKI). We assessed the ability of the following urine markers to predict AKI in patients who received amphotericin B: urine neutrophil gelatinase-associated lipocalin (NGAL), cystatin C (CysC), tissue inhibitor of metalloproteinases-2 (TIMP-2), and protein.

**Methods:**

One hundred and thirty human immunodeficiency virus (HIV)–infected participants with cryptococcal meningitis were enrolled and received amphotericin and fluconazole for 2 weeks. We defined AKI as glomerular filtration rate (GFR) < 60 mL/min/1.73 m^2^; measured urine NGAL, CysC, TIMP-2, and protein; and explored AKI incidence, risk factors, and associations with mortality using Cox proportional hazards models.

**Results:**

Participants were 48% female with a median age of 35 years, a median CD4 count of 21 cells/μL, and 44% died within 12 months. Incident AKI occurred in 42% and was associated with mortality (adjusted hazard ratio [aHR] = 2.8; *P* < .001). Development of AKI was associated with female sex (*P* = .04) and with higher CD4 count (49 vs 14 cells/μL; *P* < .01). Urine protein level in the highest quartile independently predicted AKI and mortality (aHR = 1.64, *P* = .04; aHR = 2.13, *P* = .02, respectively). Urine NGAL levels in the highest quartile independently predicted AKI (aHR = 1.65; *P* = .04).

**Conclusions:**

Acute kidney injury occurred in 42% of patients, and AKI was associated with mortality. Urine biomarkers, specifically urine protein, may be useful for antecedent prediction of amphotericin-associated AKI but need further evaluation.

## Summary

Acute kidney injury (AKI) occurs commonly in human immunodeficiency virus–infected patients with cryptococcal meningitis treated with 2 weeks of amphotericin B deoxycholate and is associated with mortality. Urine protein may play a role in earlier diagnosis of AKI but needs further evaluation.

Cryptococcal meningitis is a disseminated fungal infection caused by *Cryptococcus* species and remains a major cause of mortality in human immunodeficiency virus (HIV)–infected persons, especially in resource-limited settings [1]. Cryptococcosis causes approximately 2% of deaths of HIV-infected persons in the United States [2] and approximately 15% of AIDS-related deaths in sub-Saharan Africa [1, 3, 4]. Liposomal amphotericin formulations remain unavailable in resource-limited settings due to cost; thus amphotericin B deoxycholate (hereafter amphotericin) remains the key treat ment for cryptococcosis despite numerous toxicities. The most common and severe side effect of amphotericin is renal toxicity, which has been reported in 49%–65% of patients [5]. Renal toxicity is caused through a combination of mechanisms, which are incompletely understood. First, systemic and renal afferent arteriolar vasoconstriction decrease kidney perfusion [6], and the resulting decrease in glomerular filtration rate (GFR) may be mitigated by aggressive saline hydration [7, 8]. Second, regardless of hydration status, direct cumulative damage to renal tubular cells occurs from induction of proinflammatory cytokines, channel formation across renal cell membranes, and apoptosis [9, 10].

Acute kidney injury (AKI) is a clinical syndrome, which has wide etiologies and was defined, staged, and classified in 2012 [11]. Acute kidney injury predicts mortality, independent of underlying disease [12], and AKI is a common complication of HIV infection [13, 14]. Definition and staging of AKI depends on serum creatinine or urine output [11], which are both late indicators of kidney injury. This has led to a wide interest in new urine and serum biomarkers that could detect AKI earlier in critically ill patients [15]. Neutrophil gelatinase-associated lipocalin (NGAL; also known as Lipocalin-2) is a 25-kDa protein produced in many tissues, including renal epithelial cells, and is an established sensitive early marker of acute ischemic kidney injury [16, 17]. Elevated urine NGAL levels predict a composite outcome of dialysis initiation or death during hospitalization when used as a risk stratification tool in unselected emergency department patients at the time of admission to the hospital [18]. Cystatin C (CysC), a 13-kDa proteinase inhibitor, can be measured in serum and in urine and, unlike creatinine, is not affected by muscle mass or age [19]. Urine CysC is a marker of tubular dysfunction or damage [20], and elevated levels may be associated with AKI [15]. Tissue inhibitor of metalloproteinases-2 (TIMP-2) is an inducer of G1 cell-cycle arrest, predicts severe AKI, and is associated with mortality in critically ill adult patients [21]. Neutrophil gelatinase-associated lipocalin and CysC are induced during inflammation. These biomarkers have not been evaluated in the context of HIV infection and opportunistic infections. In resource-limited settings where liposomal amphotericin is not available, a urine biomarker that could predict development of AKI early and accurately could be a valuable clinical tool to tailor duration of amphotericin therapy.

We assessed these urine biomarkers together with urine protein and creatinine levels in patients with HIV-associated cryptococcal meningitis treated with amphotericin therapy and investigated association with AKI and mortality.

## METHODS

Human immunodeficiency virus–infected, antiretroviral therapy (ART)–naive adults with a new diagnosis of cryptococcal meningitis were prospectively enrolled into the Cryptococcal Optimal ART Timing (COAT) trial during 2010–2012 and treated with amphotericin B, fluconazole, and ART according to the COAT trial protocol [22, 23]. Baseline and follow-up serum creatinine concentrations were measured at regular intervals as per the COAT trial protocol (5–6 time points during amphotericin treatment and at 1 and 2 weeks after completion of amphotericin therapy). Estimated GFR was calculated using the Modification of Diet in Renal Disease (MDRD) study equation [24], and AKI was defined as a decrease in GFR to <60 mL/min/1.73 m^2^ at any point within 3 weeks of cryptococcal diagnosis. Urine output was not routinely monitored and could not be used to define AKI. Ten-week and 12-month mortality was ascertained.

We included 130 participants with stored urine samples, which were collected a median of 4 (interquartile range [IQR] = 4–5) days from diagnosis of cryptococcal meningitis (which is similar to time from amphotericin initiation: median = 4; IQR = 4–5 days), and measured NGAL, CysC, and TIMP-2 by enzyme-linked immunosorbent assay (R&D Systems) and protein and creatinine (Beckman Coulter DXC 800) on stored urine samples. We summarized incidence of AKI and explored risk factors for incident AKI using Cox univariate and multivariable proportional hazards models. Models were estimated with biomarkers on a continuous scale (after log_2_ transformation, so that the hazard ratio is per doubling of the biomarker). Other models compared the upper quartile versus the lower 3 quartiles (an a priori comparison to assess clinical outliers). Each biomarker was first statistically tested individually and then in a multivariable model adjusted for timing of ART initiation group, age, sex, altered mental status at diagnosis, CD4 cell count, and cerebrospinal fluid quantitative cryptococcal culture at diagnosis. We examined the association of incident AKI with 12-month mortality using time-updated Cox proportional hazards models. With the time-updated models, an indicator for incident AKI was recalculated at the time of each creatinine measurement to assess the impact of developing AKI on mortality. All analyses were completed using SAS 9.3. Relevant ethics and regulatory authority approvals were obtained. Written informed consent was obtained to participate in the COAT trial, as previously described [22].

## RESULTS

### Incidence and Risk of Acute Kidney Injury

The COAT trial enrolled 177 patients [22], of whom 130 patients had urine samples stored and were included in this study. The median age was 35 years (IQR = 30–40), and 52% (n = 68/130) were men. The median CD4 T-cell count was 21 cells/μL (IQR = 9–74). Forty-four percent (n = 57/130) died within 12 months; 1 withdrew consent and 1 was lost to follow-up. Four persons (3%) had GFR <60 mL/min/1.73 m^2^ at baseline. Incident AKI occurred in 42% (n = 53/126) at a median of 8 days (IQR = 6–11) after diagnosis of cryptococcal meningitis. Persons developing AKI had higher baseline CD4 T-cell counts (49 vs 14 cells/μL; *P* < .01), and a higher proportion were women (59% vs 40%; *P* = .04). Table 1 displays baseline characteristics, urine biomarker values, and mortality.

**Table 1.** Urinary Biomarkers in Human Immunodeficiency Virus–Associated Cryptococcal Meningitis: Baseline Characteristics for the Overall Cohort, Participants Who Developed Acute Kidney Injury, and Those Who Did Not

Baseline Variables	Overall Cohort^a^	Incident Acute Kidney Injury	No Acute Kidney Injury	*P* Value^b^
No.	130	53	73	…
Early ART initiation arm, no. (%)	63 (49)	26 (49)	37 (51)	.92
Age, median (IQR), y	35 (30–40)	34 (30–40)	37 (29–40)	.55
Female sex, no. (%)	62 (48)	31 (59)	29 (40)	**.04**
Glasgow Coma Scale^c^ < 15, no. (%)	38 (29)	17 (32)	20 (28)	.60
CSF quantitative culture, median (IQR), log_10_ CFU/mL	5.0 (3.8–5.4)	5.1 (3.8–5.5)	5.0 (4.0–5.6)	.91
CD4 count, median (IQR), cells/μL	21 (9–74)	49 (16–87)	14 (7–54)	**<.01**
Weight^d^, median (IQR), kg	54 (46–60)	54 (47–57)	54 (45–60)	.81
Serum creatinine, median (IQR), μmol/L	70 (50–88)	69 (50–91)	70 (50–80)	.36
Serum creatinine, median (IQR), mg/dL	0.79 (0.57–1.00)	0.78 (0.57–1.03)	0.79 (0.57–0.90)	.36
Serum potassium, median (IQR), mEq/L	3.9 (3.4–4.2)	3.8 (3.3–4.1)	3.9 (3.5–4.2)	.37
Amphotericin dose^e^, mg/kg/d				
Overall	0.93 (0.85–1.00)	0.95 (0.89–1.0)	0.93 (0.83–1.0)	.62
Men	0.93 (0.81–1.0)	0.96 (0.91–1.0)	0.92 (0.81–1.0)	…
Women	0.95 (0.89–1.00)	0.94 (0.89–1.0)	0.95 (0.91–1.0)	…
Urine biomarkers^f^				
Cystatin C, ng/mL	2.2 (1.3–3.5)	2.6 (1.5–3.5)	2.0 (1.2–3.3)	.32
Neutrophil gelatinase-associated lipocalin, ng/mL	4.1 (3.0–5.7)	4.2 (3.1–6.1)	4.0 (3.0–5.0)	.41
Tissue inhibitor of metalloproteinases-2, ng/mL	1.8 (1.4–2.5)	1.9 (1.5–2.7)	1.6 (1.3–2.1)	**<.01**
Urinary creatinine, mg/dL	4.6 (3.6–5.4)	4.8 (3.3–5.7)	4.4 (3.7–5.1)	.39
Urinary protein, g/L	0.21 (0.11–0.39)	0.29 (0.16–0.46)	0.16 (0.08–0.30)	**<.01**
Protein/creatinine ratio	0.56 (0.36–0.83)	0.57 (0.30–0.79)	0.56 (0.38–0.96)	.49
Ten-week mortality, no. (%)	48 (37)	27 (51)	21 (29)	**.01**
Twelve-month mortality, no. (%)	57 (44)	29 (55)	28 (38)	**.07**

Bolded *P* values indicate statistically significant difference between patients who developed AKI and those who did not.

Abbreviations: ART, antiretroviral therapy; CFU, colony-forming unit; CSF, cerebrospinal fluid; IQR, interquartile range.

^a^Includes 4 people with prevalent estimated glomerular filtration rate <60 mL/min/1.73m^2^ at cryptococcal meningitis diagnosis.

^b^Kruskall-Wallis or χ^2^ tests as appropriate, comparing those who develop incident acute kidney injury to those who do not.

^c^Glasgow Coma Scale <15 denotes altered mental status.

^d^Weight was measured for n = 111 participants (44 of 53 who later developed incident acute kidney injury; 63 of 73 who did not develop incident acute kidney injury).

^e^Amphotericin dose (mg/kg/d) is calculated only for those with a weight measurement.

^f^Samples collected at median of 4 days on amphotericin B therapy and stored at −80°C

### Urine Biomarker Associations With Acute Kidney Injury

**Figure 1.**
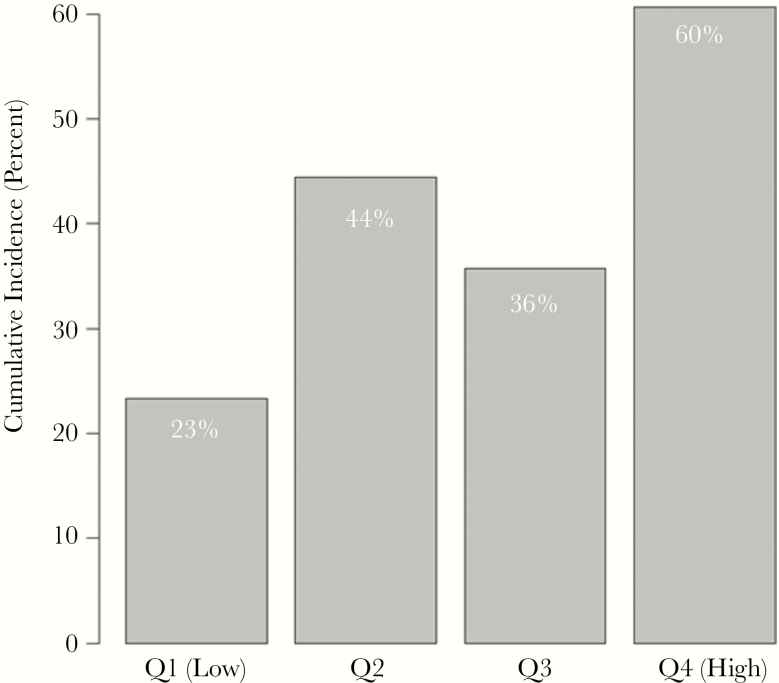
Urinary biomarkers in human immunodeficiency virus–associated cryptococcal meningitis: acute kidney injury by quartiles of urine protein values. χ^2^*P* value comparing the 4 groups = .03. Abbreviation: Q, quartile.

**Figure 2.**
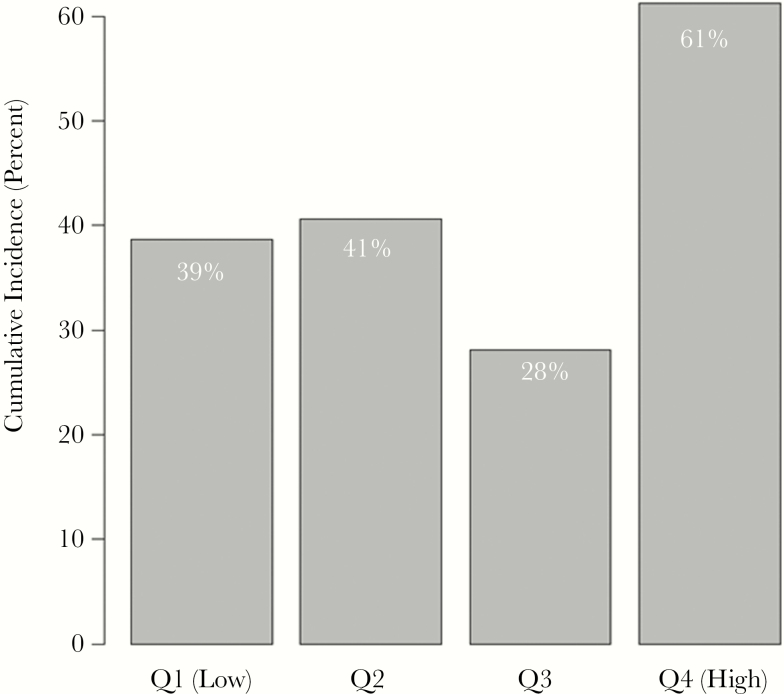
Urinary biomarkers in human immunodeficiency virus–associated cryptococcal meningitis: acute kidney injury by quartiles of urine neutrophil gelatinase-associated lipocalin (NGAL) values. χ^2^*P* value comparing the 4 groups = .06. Abbreviation: Q, quartile.

On a continuous scale, there was increased risk of AKI per doubling of urine TIMP-2 and protein levels (hazard ratio [HR] = 1.47, 95% confidence interval [CI] = 1.08–2.00, *P* = .02; HR = 2.28, 95% CI = 1.05–4.94, *P* = .04, respectively), but the association became nonsignificant after adjustment (adjusted HR [aHR] = 1.34, 95% CI = 0.95–1.88, *P* = .09; aHR = 2.23, 95% CI = 0.92–5.41, *P* = .07). In univariate models, those with NGAL, TIMP-2, and protein levels in the upper quartile had an increased risk of AKI compared with those in the lower quartiles. The risk remained significant for NGAL and protein in multivariable models. Sixty percent of those with values in the highest quartile of protein (>0.30 g/L protein) developed AKI as compared with an average of 35% of those in the lower 3 quartiles (aHR = 1.64; 95% CI = 1.02–2.62; *P* = .04) (Figure 1 and Table 2). Similarly, of those in the upper quartile of NGAL (>47.7 ng/mL), 61% developed AKI as compared with an average of 36% of those in the lower 3 quartiles (aHR = 1.65; 95% CI = 1.01–2.69) (Figure 2 and Table 2).

**Table 2.** Urinary Biomarkers in Human Immunodeficiency Virus–Associated Cryptococcal Meningitis: Urinary Biomarkers Associated With Incident Acute Kidney Injury

Urine Biomarker	Association Between Urinary Biomarkers (per doubling of value) and Acute Kidney Injury			
	Univariate Hazard Ratio^a^ (95% CI)	*P* Value	Multivariable Hazard Ratio^a^^,^^c^ (95% CI)	*P* Value
CysC	1.04 (0.88–1.23)	.62	1.05 (0.88–1.25)	.61
NGAL	1.06 (0.91–1.24)	.46	1.01 (0.85–1.19)	.91
TIMP-2	1.47 (1.08–2.00)	**.02**	1.34 (0.95–1.88)	.09
Protein	2.28 (1.05–4.94)	**.04**	2.23 (0.92–5.41)	.07
Creatinine	1.04 (0.82–1.32)	.74	1.07 (0.83–1.38)	.59
Protein/creatinine ratio	0.83 (0.38–1.80)	.63	0.63 (0.28–1.45)	.28
Association of Upper Quartile of Urinary Biomarkers With Acute Kidney Injury: Fourth Quartile of Biomarkers Compared With Quartiles 1–3				
	Hazard Ratio^b^ (95% CI)	*P* Value	Hazard Ratio^c^ (95% CI)	*P* Value
CysC	1.05 (0.70–1.59)	.81	1.05 (0.68–1.63)	.82
NGAL	1.92 (1.25–2.94)	**<.01**	1.65 (1.01–2.69)	**.04**
TIMP-2	1.70 (1.10–2.61)	**.02**	1.46 (0.91–2.33)	.12
Protein	1.73 (1.12–2.68)	**.01**	1.64 (1.02–2.62)	**.04**
Creatinine	1.41 (0.92–2.16)	.12	1.48 (0.94–2.32)	.09
Protein/creatinine ratio	0.92 (0.60–1.42)	.71	0.84 (0.53–1.34)	.47

Bolded *P* values indicate statistically significant difference between patients who developed AKI and those who did not.

Abbreviations: CI, confidence interval; CysC, cystatin C; NGAL, neutrophil gelatinase-associated lipocalin; TIMP-2, tissue inhibitor of metalloproteinases-2.

^a^Proportional hazards regression analyses using log_2_ transformed biomarker data. The hazard ratio presents the risk per doubling of the biomarker for developing incident acute kidney injury (GFR < 60 mL/min/1.73 m^2^) within 3 weeks of diagnosis of cryptococcal meningitis.

^b^Fourth quartile versus quartile 1–3. The hazard ratio presents the risk of developing acute kidney injury if the urine biomarker level falls within the highest quartile compared with the lower 3 quartiles.

^c^Adjusted for antiretroviral treatment (ART) group (early ART or deferred ART group), age, sex, decreased level of consciousness at diagnosis, CD4 cell count, and cerebrospinal fluid quantitative cryptococcal culture at diagnosis (but not for other biomarkers).

### Associations with 12-Month Mortality

**Figure 3.**
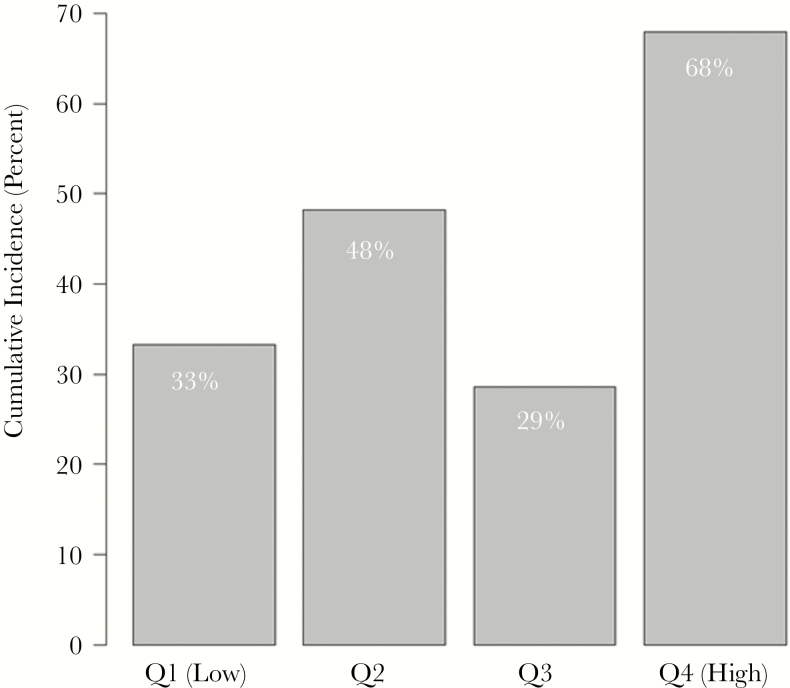
Urinary biomarkers in human immunodeficiency virus–associated cryptococcal meningitis: 12-month mortality by quartiles of urine protein values. χ^2^*P* value comparing the 4 groups = .01. Abbreviation: Q, quartile.

Elevated urine NGAL and protein-to-creatinine ratio, respectively, were associated with mortality in univariate analysis (HR = 1.16, 95% CI = 1.01–1.34, *P* = .04; HR = 1.86, 95% CI = 1.01–3.41, *P* = .05) but not in multivariable analysis (aHR = 1.14, 95% CI = 0.98–1.34, *P* = .09; aHR = 1.53, 95% CI = 0.73–3.20, *P* = .26) when evaluated on a continuous log_2_ scale. Urine protein levels in the upper quartile independently predicted 12-month mortality, and 68% of patients with urine protein levels in the highest quartile died within 12 months as compared with 23%, 44%, and 36% of those in the lower quartiles, respectively (aHR = 2.13; 95% CI = 1.15–3.96) (Figure 3 and Table 3).

**Table 3.** Urinary Biomarkers in Human Immunodeficiency Virus–Associated Cryptococcal Meningitis: Analyses of Urinary Biomarkers and Acute Kidney Injury With 12-Month Mortality

Urine Biomarker	Association Between Urinary Biomarkers (per doubling of value) and 12-Month Mortality			
	Univariate Hazard Ratio^a^ (95% CI)	*P* Value	Multivariable Hazard Ratio^a,c^ (95% CI)	*P* Value
CysC	1.15 (0.99–1.34)	.06	1.14 (0.98–1.33)	.10
NGAL	1.16 (1.01–1.34)	**.04**	1.14 (0.98–1.34)	.09
TIMP-2	1.25 (0.91–1.73)	.17	1.23 (0.87–1.75)	.24
Protein	1.92 (0.90–4.11)	.09	1.94 (0.83–4.57)	.12
Creatinine	1.08 (0.88–1.33)	.45	1.16 (0.92–1.47)	.21
Protein/creatinine ratio	1.86 (1.01–3.41)	**.05**	1.53 (0.73–3.20)	.26
Acute kidney injury^d^ (GFR < 60)	3.27 (1.87–5.73)	**<.001**	2.82 (1.55–5.17)	**<.001**
Severe acute kidney injury^d^ (GFR < 30)	12.17 (4.98–29.73)	**<.001**	8.11 (3.19–20.60)	**<.001**
Association of Upper Quartile of Urinary Biomarkers with 12-Month Mortality: Fourth Quartile of Biomarkers Compared With Quartiles 1–3				
	Hazard Ratio^b^ (95% CI)	*P* Value	Hazard Ratio^c^ (95% CI)	*P* Value
CysC	1.04 (0.57–1.87)	.91	1.18 (0.63–2.19)	.61
NGAL	1.12 (0.62–2.02)	.71	0.99 (0.52–1.88)	.97
TIMP-2	1.56 (0.85–2.85)	.15	1.66 (0.87–3.17)	.12
Protein	2.30 (1.30–4.08)	**<.01**	2.13 (1.15–3.96)	**.02**
Creatinine	1.11 (0.59–2.08)	.75	1.20 (0.61–2.35)	.59
Protein/creatinine ratio	2.03 (1.14–3.62)	**.02**	1.53 (0.80–2.93)	.20

Bolded *P* values indicate statistically significant difference between patients who died within 12 months after diagnosis of cryptococcal meningitis and those who did not.

Abbreviations: CI, confidence interval; CysC, cystatin C; GFR, estimated glomerular filtration rate using the Modification of Diet in Renal Disease study equation; NGAL, neutrophil gelatinase-associated lipocalin; TIMP-2, tissue inhibitor of metalloproteinases-2.

^a^Proportional hazards regression analyses using log_2_-transformed biomarker data. The hazard ratio presents the 12-month mortality risk per doubling of the biomarker.

^b^Fourth quartile versus quartile 1–3. The hazard ratio presents the 12-month mortality risk if the urine biomarker level falls within the highest quartile compared to the lower three quartiles.

^c^Adjusted for antiretroviral treatment (ART) group (early ART or deferred ART group), age, sex, decreased level of consciousness at diagnosis, CD4 cell count, and cerebrospinal fluid quantitative cryptococcal culture at diagnosis (but not for other biomarkers).

^d^From time-updated models with indicators for GFR <30 or <60 mL/min/1.73m^2^ at each visit.

Incident AKI was independently associated with 12-month mortality (time-updated aHR = 2.82; 95% CI = 1.55–5.17; *P* < .001). Patients developed AKI at a median of 8 days after diagnosis of cryptococcal meningitis (IQR = 6–11 days) and died a median of 8 days (IQR = 4–23 days) after AKI developed. All 9 patients who developed severe AKI (estimated GFR of <30 mL/min/1.73 m^2^) died.

### Ten-Week Outcomes in Acute Kidney Injury Patients

The outcome of abnormal renal function was assessed in survivors at 10 weeks after diagnosis of cryptococcal meningitis. Among 53 patients who developed AKI, 51% (n = 27) died before 10 weeks, and renal function recovered to eGFR >60 mL/min/1.73 m^2^ in the remaining 49% (n = 26/53) by 8 weeks. All 4 patients with abnormal renal function at baseline survived and recovered renal function to eGFR >60 mL/min/1.73 m^2^ by 10 weeks.

## DISCUSSION

To our knowledge, this is the first study to evaluate urine biomarkers in patients with HIV-associated cryptococcal meningitis treated with amphotericin. Acute kidney injury was a common complication and associated with high mortality. Urine protein level in the highest quartile independently predicted AKI and 12-month mortality, whereas the other biomarkers tested showed no strong associations. Urine biomarkers are being increasingly applied in clinical practice, particularly in settings where AKI is common and can potentially be mitigated by amending management strategies, such as intensive care units. Urine biomarkers have not been studied in the context of HIV-associated cryptococcosis where AKI is a common complication of amphotericin therapy and mortality is high. Urine protein can also be measured by urine dipstick, which is a cheap and readily available test in resource-limited settings. This could be a useful marker to investigate in similar settings as an adjunctive tool to aid clinical decision making. Elevated urine protein levels at initiation of therapy could prompt more intensive fluid replacement, closer monitoring of renal function, and possible decreased duration of amphotericin, but this needs to be evaluated in further studies.

Acute kidney injury, as defined in this study, developed in 42% of patients in this cohort. The Kidney Disease: Improving Global Outcomes (KDIGO) published case definition of AKI consists of (1) increase in serum creatinine within 48 hours of ≥26.5 μmol/L (0.3 mg/dL) or (2) increase in serum creatinine to ≥1.5 times baseline, which is known or presumed to have occurred in the prior 7 days, or urine volume of <0.5 mL/kg/h for 6 hours [11]. We did not measure urine output in this cohort, and using serial serum creatinine values, 80% of our patients met creatinine-based KDIGO AKI case definition criteria. We used an amended case definition, which is equal to a grade 3 (severe) adverse event [25] and has clinical relevance in this critically ill cohort who required treatment with a nephrotoxic drug. Amphotericin therapy is generally interrupted or stopped when a patient’s GFR declines to <60 mL/min/1.73 m^2^.

People with higher CD4 T-cell counts developed AKI more commonly in our study. The reason for this is unclear. It is possible that patients with very low CD4 T-cell counts did not survive to develop AKI, and there is thus a competing risk of death; however, CD4 count was not associated with survival in this cryptococcal cohort [22]. More women developed AKI in our study, which is consistent with the literature, which lists women as more susceptible to AKI [26].

Tissue inhibitor of metalloproteinases-2 and urine protein levels were associated with development of AKI in univariate analysis but not after adjusting for potential confounders on a continuous scale. Urine TIMP-2 combined with insulin-like growth factor-binding protein 7 (IGFBP7) has been shown to predict development of severe AKI in acutely ill patients within 12 hours of collection of urine sample [27]. Higher urine TIMP-2 has also been associated with mortality in acutely ill patients [21] but not in our study. Our urine samples were collected at a median of 4 days after diagnosis of cryptococcal meningitis, and AKI developed at a median of 8 days on therapy, which is a different scenario to the previous studies investigating the predictive value of TIMP-2.

Higher urine CysC was not associated with development of AKI or mortality in our study when evaluated on a continuous log_2_ scale or comparing the upper quartile with lower quartiles. Cystatin C is an early marker of tubular dysfunction or damage, and it is possible that (1) the mechanism of development of AKI in our cohort was different or (2) that we measured CysC levels too long before AKI events to detect elevated levels.

We found AKI to be independently associated with mortality. Patients in this study were severely immune suppressed, and although it is possible that AKI secondary to amphotericin therapy could have played a role in mortality, there are other factors such as nosocomial sepsis, opportunistic infections, and decreased level of consciousness with decreased oral intake that likely contributed to both AKI and mortality.

A limitation of our study is that we used a nonstandard case definition of AKI. This limits comparability across studies, and importantly, patients who had normal renal function at baseline would have had to experience a much larger proportional decline in renal function before being classified as an AKI case compared with patients who entered the study with a GFR close to the threshold of 60 mL/min/1.73 m^2^. Furthermore, urine was collected after initiation of amphotericin therapy, and urine biomarker findings may reflect early subclinical amphotericin-related AKI and not purely the theoretical potential to develop AKI. The timing of best measurement of urine biomarkers is unknown, and further elucidation of the change in biomarkers over time would be of interest. Ultimately, the clinical utility of urine biomarkers would be optimal if future AKI could be predicted accurately early after diagnosis to guide dosing and duration of amphotericin therapy. There is considerable interest in shortening amphotericin regimens for treatment of cryptococcal meningitis in resource-limited settings [28, 29] and a urine biomarker that could predict risk of AKI accurately and early could be valuable to guide duration of amphotericin treatment. Not all patients from the COAT trial were included because not all patients had stored urine specimens. Patients who were enrolled in the COAT trial and not included in this study had no difference in baseline CD4, HIV load, age, sex, COAT treatment arm, and cerebrospinal fluid quantitative culture (data not shown) compared with this cohort.

In conclusion, AKI occurred in 42% of HIV-infected patients treated with amphotericin B deoxycholate for cryptococcal meningitis, and AKI was associated with mortality. However, AKI was transient among survivors. Urine protein levels are easy to measure and may be useful for antecedent prediction of amphotericin-associated AKI. The utility of measuring urine protein levels for aiding decisions regarding duration of amphotericin therapy needs further evaluation.
